# The application of eXplainable artificial intelligence in studying cognition: A scoping review

**DOI:** 10.1002/ibra.12174

**Published:** 2024-09-05

**Authors:** Shakran Mahmood, Colin Teo, Jeremy Sim, Wei Zhang, Jiang Muyun, R. Bhuvana, Kejia Teo, Tseng Tsai Yeo, Jia Lu, Balazs Gulyas, Cuntai Guan

**Affiliations:** ^1^ Lee Kong Chian School of Medicine Nanyang Technological University Singapore Singapore; ^2^ Centre for Neuroimaging Research Nanyang Technological University Singapore Singapore; ^3^ Division of Neurosurgery, Department of Surgery National University Hospital Singapore Singapore; ^4^ School of Computer Science and Engineering Nanyang Technological University Singapore Singapore; ^5^ Defence Medical and Environmental Research Institute DSO National Laboratories Singapore Singapore

**Keywords:** artificial intelligence, cognition, cognitive neuroscience, eXplainable artificial intelligence, neuroscience, XAI models

## Abstract

The rapid advancement of artificial intelligence (AI) has sparked renewed discussions on its trustworthiness and the concept of eXplainable AI (XAI). Recent research in neuroscience has emphasized the relevance of XAI in studying cognition. This scoping review aims to identify and analyze various XAI methods used to study the mechanisms and features of cognitive function and dysfunction. In this study, the collected evidence is qualitatively assessed to develop an effective framework for approaching XAI in cognitive neuroscience. Based on the Joanna Briggs Institute and preferred reporting items for systematic reviews and meta‐analyses extension for scoping review guidelines, we searched for peer‐reviewed articles on MEDLINE, Embase, Web of Science, Cochrane Central Register of Controlled Trials, and Google Scholar. Two reviewers performed data screening, extraction, and thematic analysis in parallel. Twelve eligible experimental studies published in the past decade were included. The results showed that the majority (75%) focused on normal cognitive functions such as perception, social cognition, language, executive function, and memory, while others (25%) examined impaired cognition. The predominant XAI methods employed were intrinsic XAI (58.3%), followed by attribution‐based (41.7%) and example‐based (8.3%) post hoc methods. Explainability was applied at a local (66.7%) or global (33.3%) scope. The findings, predominantly correlational, were anatomical (83.3%) or nonanatomical (16.7%). In conclusion, while these XAI techniques were lauded for their predictive power, robustness, testability, and plausibility, limitations included oversimplification, confounding factors, and inconsistencies. The reviewed studies showcased the potential of XAI models while acknowledging current challenges in causality and oversimplification, particularly emphasizing the need for reproducibility.

## Graphical abstract

Experimental research in neuroscience has highlighted the significance of eXplainable artificial intelligence (XAI) in studying cognition. Cognition can be characterized by key domains such as perceptual‐motor control, social cognition, executive function, and memory. Recent research efforts have begun to address existing knowledge gaps in specific aspects of cognition or a cognitive disease by applying XAI's explanatory techniques to extensive data sets. These XAI methods, varying in effectiveness, have attempted to elucidate the underlying AI processes in identifying or modeling (patho)physiologic mechanisms and features of a particular cognitive function. This scoping review therefore broadly mapped out pertinent evidence available in the current literature on the different XAI models used in cognitive studies. Qualitative analysis was subsequently performed in a thematic fashion.

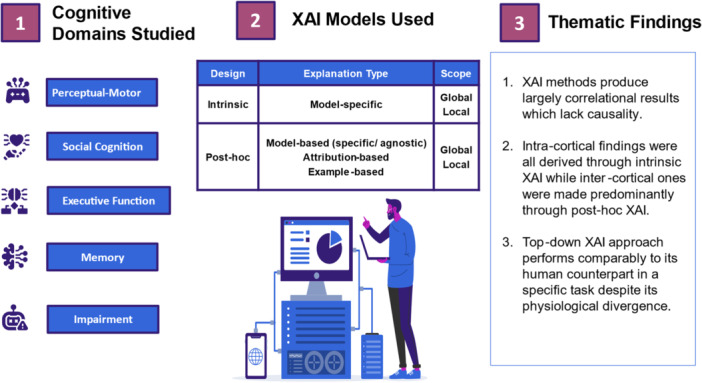

## 1 INTRODUCTION

Recent decades have documented the rapid progression of artificial intelligence (AI) in performing various cognitive tasks which would otherwise necessitate human intelligence.^1^ AI has not only pervaded day‐to‐day life^2^ but also promised far‐reaching advances in a myriad of industries.^3^ Current examples that illustrate its ubiquity and significance include the development and application of AI systems^4^ in agriculture, finance, transport, retail, sports, and healthcare— just to name a few. The fierce and massive burgeoning of AI has precipitated studies on its trustworthiness, which has been viewed as the crux of the technology's value.^5^ Of note, this inquiry into trustworthiness has reinvigorated scholarly discussion on the decades‐long concept of eXplainable AI (XAI).^6^, ^7^, ^8^ In other words, the AI community at present is pursuing explainability as one of the defining features of a trustworthy AI.^9^ This has been, by extension, illustrated in a recent survey by Tjoa and Guan^10^ which shed light on the utility of XAI, particularly in the clinical setting where reliability is indispensable.

**Figure 1:**
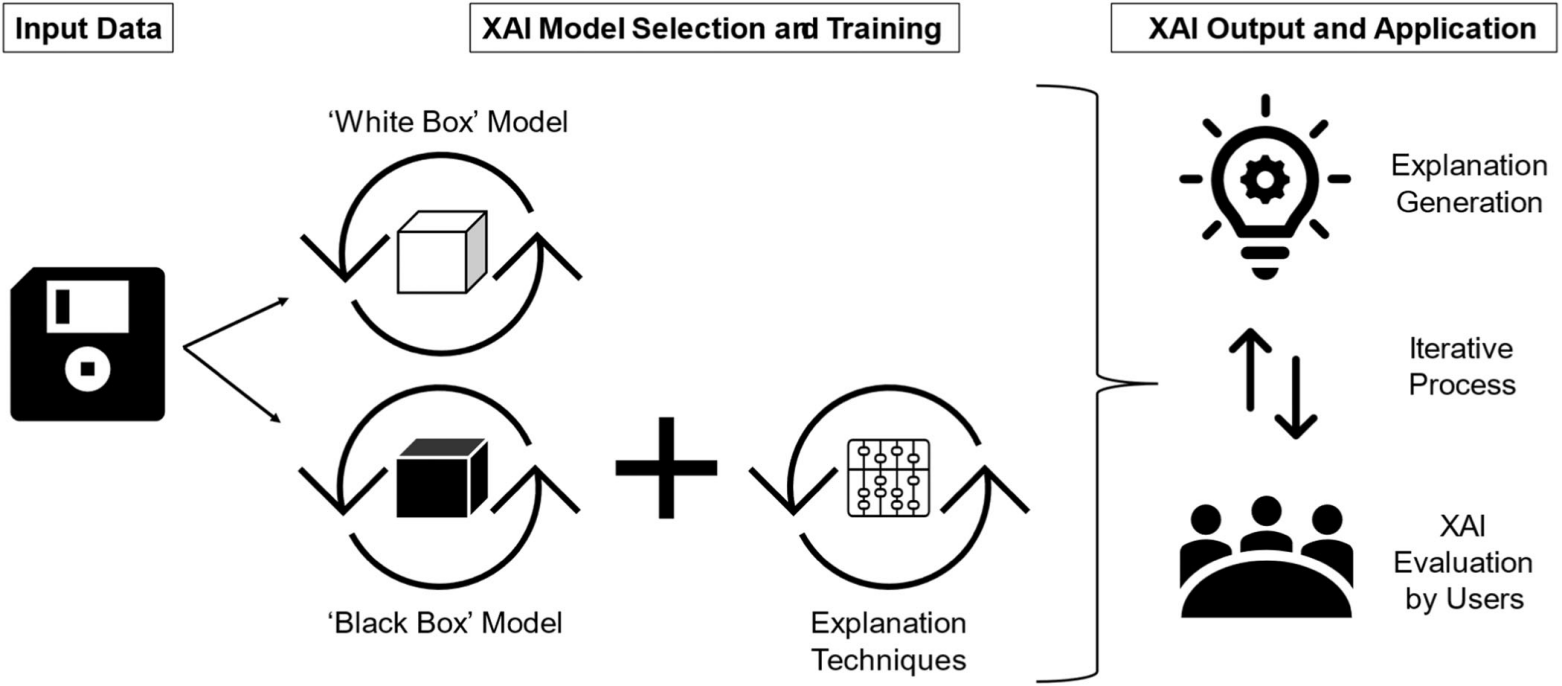
Typical workflow in XAI development and application. This diagram is a simplified pictorial representation of the typical workflow in XAI development and application. The process involves input data, XAI model selection, and training, followed by iterative explanation generation (^9^, ^11^). Descriptive details can be found in Table 1. XAI, eXplainable artificial intelligence.

The XAI terminology, however, lacks established criteria^10^, ^11^ as studies tend to take a more intuitive approach toward the concept of explainability.^12^ Nonetheless, as informed by the current literature, there are common salient features which underpin XAI.^13^ In particular, interpretability (how understandable an explanation is for humans) and fidelity (the accuracy of an explanation in describing the task model behavior) are necessary for deeming an AI tool explainable.^14^ Table 1 summarizes the key stages in the development of an XAI while Figure 1 illustrates its typical workflow.

**Table 1:** Stages in the Development of an XAI Model.

XAI development	Symbol	Description
Stage 1: Input data	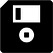	Collected data sets are fed into the chosen XAI model.
Stage 2: XAI model selection (refer to Table 3)	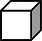	Intrinsic or “white box” XAI models are explainable by design where the inner mechanism is transparent to users.
	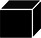	“Black box” models are opaque and comprise the majority of AI. To achieve a certain degree of explainability, XAI explanation techniques (stage 3) are coupled with the chosen “black box” model.
Stage 3: Explanation technique selection	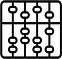	Refer to Table 3 for the different types of explanation techniques.
Stage 4: XAI model training	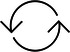	Train the XAI model using the selected data and explanation techniques. Assess the model's accuracy and fidelity using appropriate metrics and validation methods.
Stage 5: Explanation generation	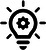	Generate explanations for the XAI model's decisions. These explanations should be interpretable, i.e., understandable to users and should provide insight into how the model works and why it made specific predictions.
Stage 6: XAI evaluation by users	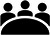	Experts in their respective scientific fields can extract relevant explanations from the XAI model to corroborate or discover novel real‐world concepts.
Stage 7: XAI model iteration	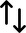	Spurious explanations and/or data input can be sieved out iteratively to optimize the XAI model's performance.

Abbreviation: XAI, eXplainable artificial intelligence.

Among its many potential applications, the new wave of experimental research in neuroscience has highlighted XAI's relevance in studying cognition.^15^ Cognition comprises various cognitive domains such as complex attention, executive function, learning and memory, language, perceptual‐motor control, and social cognition.^16^, ^17^ Recent research efforts have offered possible inroads into plugging the knowledge gaps in specific aspects of cognition or a cognitive disease by employing the explanatory techniques of XAI in processing pools of collected data.^18^ These XAI techniques, at varying degrees of effectiveness, have provided an understanding of the underlying AI processes in identifying or even modeling key physiologic or pathophysiologic mechanisms and features of a particular cognitive function. It is therefore imperative to explore XAI's potential influence on studying cognition by broadly mapping out the pertinent evidence available in the current literature.

Previous reviews on XAI were largely descriptive and broadly concerned with the XAI definition, choices of design models, and evaluative methods, irrespective of discipline.^19^ However, to the best of our knowledge, the application of XAI in cognitive neuroscience is still currently a novel concept^20^, ^21^, ^22^ and no review has been published in this area. This scoping review aimed to provide a macroscopic viewpoint on the different XAI methods used particularly in studying both cognitive function and dysfunction. Subsequently, the evidence presented was then assessed qualitatively, thereby allowing for the formulation of a viable framework in approaching XAI in the context of cognitive neuroscience.

In this study, our objective was to (1) explore the cognitive functions and dysfunctions studied through the application of XAI, (2) identify the different methods of XAI applied in cognitive research, and (3) uncover the salient findings from these applications. Additionally, we aimed to (4) assess the strengths of XAI, or reasons for its use, in studying cognition, as well as (5) the associated challenges and limitations.

## 2 METHODS

### 2.1 Study design

This scoping review aimed to explore the breadth of the literature, map the evidence, and inform future research.^23^ Certain components typically expected of a systematic review, such as critical appraisal (quality and risk‐of‐bias assessment) of included studies and data synthesis (qualitative or statistical meta‐analysis, or mixed‐methods), are beyond the design of a scoping review.^24^

The scoping review methodology was chosen given the absence of a review broadly overviewing XAI application within the context of cognition. We followed the Joanna Briggs Institute^23^ and the Preferred Reporting Items for Systematic Reviews and Meta‐Analyses extension for Scoping Reviews (PRISMA‐ScR) reporting guidelines (Appendix A).^25^

### 2.2 Search strategy

The search strategy was developed iteratively to cover related words defining “explainable artificial intelligence” and “cognition.” The search was performed on January 20, 2023, which included the following peer‐reviewed databases and gray literature:

- MEDLINE (Ovid)
- Embase
- Cochrane Central Register of Controlled Trials (CENTRAL)
- Web of Science
- First 10 pages of Google Scholar

The reference lists of included studies and other reviews on XAI were also examined to identify additional included studies.

### 2.3 Screening of studies

All retrieved studies were uploaded to EndNote 20 and screened on Covidence. The screening was based on the eligibility criteria outlined in Table 2. The title‐and‐abstract and full‐text screening were performed by two independent reviewers in parallel. Discrepancies in either stage were resolved by discussion between the reviewers.

**Table 2:** Inclusion and exclusion criteria.

Inclusion criteria	Exclusion criteria
The use of XAI in understanding the underlying mechanisms of cognitive function(s) and/or disease(s). The use of XAI in understanding the underlying features of cognitive function(s) and/or disease(s). The use of established, modified, and/or novel XAI model(s). Published between January 2013 and January 2023. Printed in English.	The use of XAI for the sole purpose of detecting, classifying, and/or predicting cognitive function(s) and/or disease(s) without giving insights into the underlying mechanism(s) and/or feature(s). The use of XAI in studying the brain without specifically mentioning cognitive function(s) and/or disease(s). Unavailable full text. Editorials, opinion pieces, and poster or conference presentations.

Abbreviation: XAI, eXplainable artificial intelligence.

### 2.4 Selection of studies

To capture data arising from the recent proliferation of XAI research,^18^ studies published between January 2013 and January 2023 were included. This review focused on the application of XAI methods which offered insights into the underlying mechanisms and features of cognitive functions or diseases (Table 2). Studies which did not contribute to this end were excluded.

### 2.5 Data extraction

A data extraction form was developed as detailed in Appendix B. The form was piloted on three studies and was amended accordingly to ensure consistent data extraction of the remaining papers before thematic analysis. Data extraction was performed in parallel by Shakran Mahmood and Colin Teo. Consensus was sought through discussion when discrepancies in data extraction occurred.

### 2.6 Presentation of results

The search and screening processes were presented in the Preferred Reporting Items for Systematic Reviews and Meta‐Analyses (PRISMA) flowchart.^26^ The extracted data were depicted in diagrams and tables while an accompanying narrative summary covered the included studies' characteristics and key findings. The collated data included cognitive functions and impairments studied, types and findings of XAI used, as well as its strengths and limitations. Thematic analysis was subsequently done in parallel by Shakran Mahmood and Colin Teo, and the data were then organized into categories.

### 2.7 Proposed XAI taxonomy

We offered a concise overview of the proposed XAI taxonomy (Table 3), adapted from recent reviews which suggested different ways of classifying XAI.^14^, ^27^ This adapted XAI taxonomy aims to illustrate the relevant dichotomies and parameters that had been considered in selecting a specific type of XAI for studying cognition. The first dichotomy separates XAI by design. This means XAI can first be divided into intrinsically explainable and post hoc explainable models. To attain intrinsic explainability, the XAI model is, by design or structurally, self‐explanatory. It is also known as a white‐box model.^28^ This category of XAI models includes decision trees, rule‐based models, and linear models.^28^ On the other hand, the explainability of the post hoc XAI lies in its ability to explain a particular black‐box AI task model.^29^

**Table 3:** Overview of the proposed XAI taxonomy.

	Design	Explanation type	Scope
XAI method taxonomy	Intrinsic	Model (specific)	Global or local
	Post hoc	Model (specific/agnostic)	Global or local
		Attribution	
		Example	

Abbreviation: XAI, eXplainable artificial intelligence.

The second dichotomy separates XAI by the type of explanations it uses, ranging from model‐specific, model‐agnostic, attribution to example‐based explanations.^14^ Model‐based explanations comprise all techniques that utilize a model to explain the task model. Such explanations fit into the intrinsic and post hoc XAI design as the XAI model itself can be used as an explanation (intrinsic) or a separate, surrogate XAI is used to decipher and learn the underlying decision rules of the task model at hand (post hoc). If the XAI can only explain one model, it is model‐specific; if it is built to explain more than one task model, it is model‐agnostic.^30^ Thus, the explainability of intrinsic XAI is model‐specific as it is the explanation in and of itself, and has to be trained only to suit a specific task.^31^ In contrast, post hoc XAI can either be model‐specific or agnostic. Additionally, post hoc XAI can also be driven by attribution‐type explanations which attempt to quantify the explanatory power or influence of input features on the given output. Example‐based explanations are also compatible with the post hoc XAI. The technique involves identifying poorly predicted and well‐predicted instances and counterfactuals^14^ to formulate an explanation.

The third dichotomy helps to define the scope^31^ of the XAI in question. The XAI has a local scope if it only explains a particular prediction by identifying the underlying relations between specific output and input. The scope is considered global if the XAI attempts to explain the entire model by unpacking the workings of its structures and parameters. Both intrinsic and post hoc XAI can either have global or local explainability which helps to enhance trust in the overall model or a particular prediction, respectively.

## 3 RESULTS

### 3.1 Search results

**Figure 2:**
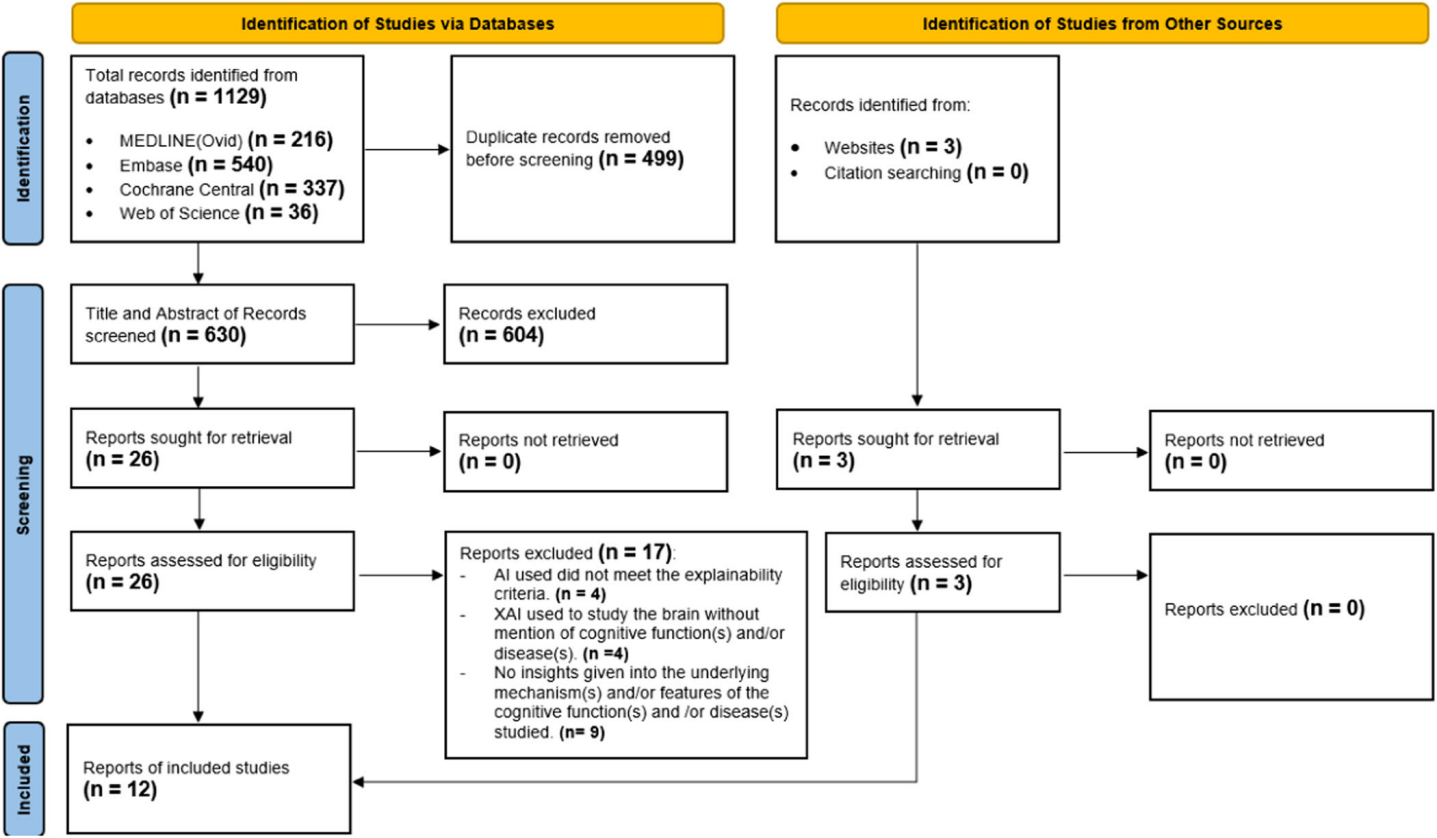
Study selection PRISMA flowchart. The study selection PRISMA flowchart illustrates the number of studies that were identified, screened, and eventually included. [Color figure can be viewed at wileyonlinelibrary.com]

As reflected in the PRISMA flowchart (Figure 2), the database search yielded 630 studies after deduplication, of which 26 full texts were retrieved. A total of 12 studies were eventually included —nine from the databases and three from the gray literature.

### 3.2 General characteristics of included studies

As can be seen in Table 4, five studies came from the United States of America, namely, Atlanta,^36^ Massachusetts,^32^, ^33^ and Texas.^34^, ^35^ Another two studies were from Tokyo, Japan.^41^, ^42^ Colchester (United Kingdom),^37^ Mons (Belgium),^38^ Reggio Calabria (Italy),^39^ Beirut (Lebanon),^40^ and Singapore^43^ had one study each. All 12 studies were experimental by design. Nine studies were published in the last 5 years while the other three were published between 2014 and 2018.

**Table 4:** General characteristics of the included studies.

Characteristics of included studies	Number of studies, *n* (%)	Study reference
Study location		
United States of America	5 (41.7%)	[33, 34, 35, 36, 37]
United Kingdom	1 (8.3%)	[38]
Belgium	1 (8.3%)	[39]
Italy	1 (8.3%)	[40]
Lebanon	1 (8.3%)	[41]
Japan	2 (16.7%)	[42, 43]
Singapore	1 (8.3%)	[44]
Study design		
Experimental	12 (100%)	[33, 34, 35, 36, 37, 38, 39, 40, 41, 42, 43, 44]
Publication year		
2013	‐	‐
2014–2018	3 (25%)	[33, 34, 43]
2019–2023	9 (75%)	[35, 36, 37, 38, 39, 40, 41, 42, 44]

### 3.3 Cognitive functions and impairments studied

Of the 12 papers as reflected in Table 5, nine (75%) studied normal cognitive functions^32^, ^33^, ^34^, ^35^, ^36^, ^37^, ^41^, ^42^, ^43^ while three (25%) studied states of impaired cognition.^38^, ^39^, ^40^ The normal cognitive functions examined by these nine papers spanned across perceptual‐motor (*n* = 4, 33.3%),^32^, ^33^, ^34^, ^37^ social cognition (*n* = 2, 16.7%),^41^, ^43^ language (*n* = 3, 25%),^35^, ^41^, ^43^ executive function (*n* = 2, 16.7%),^36^, ^42^ and memory (*n* = 1, 8.3%)^36^ domains. One (8.3%) study^37^ individually covered two cognitive functions under a single domain while another three (25%) studies^36^, ^41^, ^43^ addressed two domains each.

**Table 5:** Summary of cognitive functions and impairments studied.

Study reference	Cognitive domain	Cognitive function/impairment studied	Details of study
[33]	Perceptual‐motor	Visual perception	Studied inferior temporal neural responses through various visual recognition tasks.
[38]	Perceptual‐motor	Visual and auditory perception	Studied the activated interaction patterns between brain regions in response to auditory and visual stimuli in 6‐month‐old infants.
[34]	Perceptual‐motor	Auditory perception	Studied the influence of simple recognition tasks on the cortical auditory system.
[35]	Perceptual‐motor	Perceptual‐motor coordination	Studied the relationship between the brain and facial muscle activity.
[42]	Social cognition; Language	Recognition of emotions; Receptive language	Studied temperament estimation through child‐robot interaction.
[44]	Social cognition; Language	Recognition of emotions; receptive language	Studied the perception of emotions through speech.
[36]	Language	Receptive language	Studied cortical representations of semantic meaning with the aid of functional magnetic resonance imaging (fMRI).
[43]	Executive function	Response to feedback	Studied the appetitive and aversive specialization of the basal ganglia pathways.
[37]	Executive function; Memory	Decision‐making; working memory; recall	Studied key brain networks in decision‐making and memory through cognitive text matrices.
[41]	Not specified	Seizure	Studied electroencephalography connectivity features to analyze and identify seizure and nonseizure activity.
[39]	Not specified	Autism spectrum disorder (ASD)	Studied the structural and functional activities of the brain in ASD and neurotypical subjects.
[40]	Not specified	Mild cognitive impairment (MCI); Alzheimer's disease (AD)	Studied the progression of MCI to AD.

Under the perceptual‐motor domain, visual perception (*n* = 2, 16.7%),^32^, ^37^ auditory perception (*n* = 2, 16.7%),^33^, ^37^ and perceptual‐motor coordination (*n* = 1, 8.3%)^34^ were studied. For social cognition, both studies (*n* = 2, 16.7%)^41^, ^43^ investigated the function of recognizing emotions. The three (25%) studies, relevant to the language domain,^35^, ^41^, ^43^ focused on the receptive aspect of language. Under executive function, one study examined response to feedback^42^ while another reported on decision‐making and working memory.^36^ Pertaining to the memory domain, one (8.3%) study assessed the function of recall.^36^

The states of cognitive impairment studied were seizures,^40^ autism spectrum disorder,^38^ and the conversion from mild cognitive impairment to Alzheimer's disease^39^—each was examined by one (8.3%) study, respectively. The three studies, however, did not explicitly refer to any of the cognitive domains.

### 3.4 Choice of XAI methods

By design, the intrinsic type (*n* = 7, 58.3%)^32^, ^33^, ^35^, ^36^, ^37^, ^38^, ^42^ is the slightly more common choice compared to the post hoc type (*n* = 6, 50%).^34^, ^36^, ^39^, ^40^, ^41^, ^43^ Of note, one (8.3%) study^36^ used two XAI methods concurrently—one intrinsic and one post hoc. The intrinsic XAI methods used were computational models (*n* = 3, 25%),^32^, ^33^, ^42^ linear regression (*n* = 2, 16.7%),^35^, ^36^ multi‐variate pattern analysis (*n* = 1, 8.3%),^37^ and classical decision tree (*n* = 1, 8.3%).^38^ The post hoc XAI methods were diverse with studies utilizing the SHapley Additive exPlanations (SHAP) model (*n* = 2, 16.7%),^34^, ^41^ the Relatable Explainable Network (RexNet) model (*n* = 1, 8.3%),^43^ the Global Permutation Percent Change (G2PC) feature importance model (*n* = 1, 8.3%),^36^ the Gradient‐weighted Class Activation Mapping (Grad‐CAM) model (*n* = 1, 8.3%),^39^ and generic input‐based explanation driver methods (*n* = 1, 8.3%)^40^ (Table 6).

**Table 6:** Summary of findings from the application of XAI.

Study reference	XAI method				Findings from the application of XAI	Type of findings
	Specific name	Design	Explanation type	Scope		
[33]	Hierarchical computational model	Intrinsic	Model (specific)	Global	The output layer of the model was found to have a strong predictive power for neural responses in the IT cortex, while the middle layers were highly predictive of neural responses in the V4 area. These results suggest that the top‐down performance constraints have a direct impact on the development of intermediate visual representations.	Anatomical correlational top‐down
[38]	XAI‐powered multivariate pattern analysis for fNIRS data (xMVPA)	Intrinsic	Model (specific)	Local	xMVPA model identified patterns of brain activity (P1 and P2) in response to a dynamic visual stimulus presented to 6‐month‐old infants. The xMVPA patterns revealed the interconnectedness of cortical channel 1 with other channels, such as channel 2 and channel 4 in P1. This provided insights into a network of cortical regions involved in visual processing, including activation of the occipital and prefrontal cortex, as well as partial activation of the temporal cortex.	Anatomical correlational bottom‐up
[34]	Hierarchical computational model	Intrinsic	Model (specific)	Global	The model made better predictions of cortical responses in both primary and nonprimary auditory cortex compared to the standard spectrotemporal filter model. The improved predictions in primary regions suggest that everyday recognition tasks may impose strong constraints on the sensory system.	Anatomical correlational top‐down
[35]	SHapley Additive exPlanations (SHAP)	Post hoc	Attribution	Local	Left temporal, right frontal brain activity, coupled with both upper and lower facial microexpression, anticipate stuttering.	Anatomical correlational Bottom‐up
[42]	SHAP	Post hoc	Attribution	Local	The trained support vector regression (SVM) model was analyzed using SHAP to determine how the model utilized features for temperament estimation. SHAP values were used to measure the contribution of each feature to a particular temperament. The analysis showed that the SVM was able to learn the interaction tendencies defined in the temperament scale.	Nonanatomical correlational bottom‐up
[44]	Relatable explainable network (RexNet)	Post‐hoc	Example	Local	RexNet offers accurate and relevant explanations (saliency, contrastive cues, counterfactual synthetics), which reflected how users naturally perceive and infer vocal emotions.	Nonanatomical correlational bottom‐up
[36]	Linear regression model	Intrinsic	Model (specific)	Local	The model achieved good performance by using responses from multiple cortical regions, which suggests that various parts of the cortex have some representation of language meaning. Additionally, the study successfully decoded continuous language using only the association and prefrontal networks, without any information from the classical language network, except to detect new words. These findings highlight the importance of bilateral domain‐general semantic regions in representing natural language.	Anatomical correlational bottom‐up
[43]	Computational neural‐circuit model	Intrinsic	Model (specific)	Local	The model revealed that direct and indirect medium spiny neurons (dMSNs and iMSNs) likely have opposite effects on dopamine neurons, leading to up‐ or downregulation, respectively. This suggests that d‐block or i‐block treatments would result in a negative or positive shift of reward prediction error (RPE). Additionally, the study supported the idea that the direct and indirect pathways of the basal ganglia are essential for appetitive and aversive learning.	Anatomical causal bottom‐up
[37]	Global permutation percent change feature importance; linear regression‐ elastic net regularization	Post hoc; intrinsic	Attribution; model (specific)	Global	The methods used in combination were able to identify bias towards larger networks and provided more insight into the reasons why each brain network is important.	Anatomical correlational bottom‐up
[41]	Input‐based explanation driver methods	Post hoc	Attribution	Local	The EEG feature relevance plot is unique to each patient, suggesting that the relevance and weight assigned to each feature are also unique. This supports the observation that electroencephalography (EEG) patterns in seizure patients exhibit high variability across patients.	Anatomical absent bottom‐up
[39]	Classification decision tree	Intrinsic	Model (specific)	Local	The analysis of the predictive markers revealed the influence of the frontal and temporal lobes in the diagnosis of the disorder, which is consistent with previous findings in the literature.	Anatomical correlational bottom‐up
[40]	Gradient‐weighted class activation mapping (Grad‐CAM)	Post hoc	Attribution	Global	The xAI analysis showed that the main information is found in the delta sub‐band and that the highest relevant areas are the left‐temporal and central‐frontal lobe, the parietal lobe, the left‐frontal lobe, and the left‐frontotemporal region for 4 different subjects, respectively.	Anatomical correlational bottom‐up

Abbreviation: XAI, eXplainable artificial intelligence.

As for the explanation techniques, the intrinsic XAI methods used in all seven (58.3%) studies^32^, ^33^, ^35^, ^36^, ^37^, ^38^, ^42^ were model‐specific, in line with the proposed XAI method taxonomy. Five (41.7%) out of the six post hoc studies were attribution‐based^34^, ^36^, ^39^, ^40^, ^41^ while the remaining one (8.3%) study used example‐based explanations.^43^ Interestingly, none of the included studies utilized model‐based post hoc methods. With regard to the scope of explainability, four (33.3%) studies used XAI methods with global scope.^32^, ^33^, ^36^, ^39^ Among these four studies, two (16.7%) studies utilized intrinsic XAI methods, particularly, hierarchical computational models^32^, ^33^ while the other two (16.7%) studies applied post hoc methods.^36^, ^39^ The other eight (66.7%) studies were based on XAI methods with a local scope (Table 6).^34^, ^35^, ^37^, ^38^, ^40^, ^41^, ^42^, ^43^

### 3.5 Findings from the application of XAI

We identified common themes across the findings of all 12 studies collated in Table 6 **.** The findings can be categorized based on their focus (anatomical or nonanatomical), the type of relationship between input and output (correlational or causal), and the study approach taken (top‐down or bottom‐up). The findings with an anatomical focus can be distinguished into two levels – neuronal or cortical. As for the latter, conclusions made could be further subdivided into intracortical and intercortical levels.

Of the 12 studies, 10 (83.3%) had anatomical findings.^32^, ^33^, ^34^, ^35^, ^36^, ^37^, ^38^, ^39^, ^40^, ^42^ Only one (8.3%) of these studies^42^ had findings at the neuronal level, which posited a causal relationship between basal ganglia pathways and appetitive‐aversive learning. This study utilized intrinsic XAI, specifically the computational method. The other nine (75%) studies^32^, ^33^, ^34^, ^35^, ^36^, ^37^, ^38^, ^39^, ^40^ all reported correlational findings at the cortical level. Of these nine studies, the study on electroencephalography (EEG) seizure detection (*n* = 1, 8.3%)^40^ had reported heterogeneous results such that a clear input–output relationship was absent. The other eight studies were evenly split: four (33.3%) produced findings at the intracortical level,^32^, ^33^, ^35^, ^37^ all through the application of intrinsic XAI, while the other four (33.3%) reported on intercortical associations^34^, ^36^, ^38^, ^39^—with post hoc XAI (*n* = 3, 25%)^34^, ^36^, ^39^ more commonly used than the intrinsic XAI (*n* = 1, 8.3%).^38^

Two (16.7%) studies^41^, ^43^ had correlational findings of nonanatomical focus. Both studies examined social cognition and language, particularly about the associations involved in emotion recognition and receptive language. These two studies employed post hoc XAI—one was attribution‐based (*n* = 1, 8.3%)^41^ while the other was example‐based (*n* = 1, 8.3%).^43^

As for the approach, two (16.7%) studies^32^, ^33^ took the top‐down approach by applying external constraints to shape and optimize the eventual XAI performance. Both studies were driven by intrinsic XAI, specifically the hierarchical computational methods. The other 10 (83.3%) studies^34^, ^35^, ^36^, ^37^, ^38^, ^39^, ^40^, ^41^, ^42^, ^43^ took the bottom‐up approach by building up the XAI from theory‐based or biologically aligned components.

### 3.6 Reasons for XAI use in studying cognition

**Figure 3:**
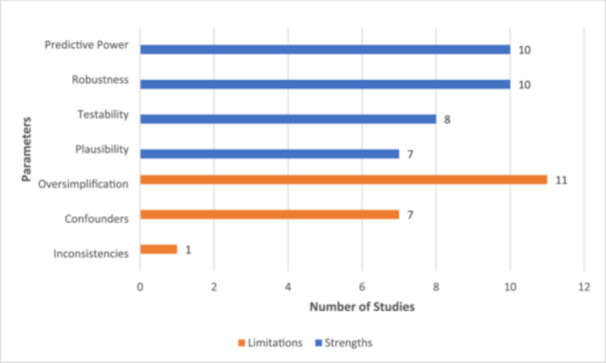
Strengths and limitations of XAI used. This horizontal bar graph depicts the strengths and limitations of XAI used in studying cognition. Predictive power and robustness were the most cited reason for XAI usage (*n* = 10, 83.3%) (33–35,37–43) while oversimplification was the most common limitation reported (*n* = 11, 91.7%) (33–41,43,44). XAI, eXplainable artificial intelligence. [Color figure can be viewed at wileyonlinelibrary.com]

Four common features or reasons were identified in favor of XAI use in studying cognition (Figure 3). The most frequently reported reason was XAI's purported predictive power and robustness (*n* = 10, 83.3%).^32^, ^33^, ^35^, ^36^, ^37^, ^38^, ^40^, ^41^, ^42^, ^43^ The claim on predictive power and robustness shared a common justification: the findings derived from the chosen XAI model corroborated established theories and past studies in the literature. Notably, two included studies went beyond these justifications by proving the statistical significance of their XAI findings (*n* = 2, 16.7%),^37^, ^41^ lending weight to the conclusion that the chosen XAI was sufficiently robust and had predictive power.

Eight (66.7%)^32^, ^33^, ^34^, ^35^, ^36^, ^37^, ^42^, ^43^ out of the 12 studies showed that their XAI methods were testable, another reason for using XAI. The testability of the XAI's findings was evaluated by manipulating variables, such as reversal learning of the given task (*n* = 8, 66.7%).^32^, ^33^, ^34^, ^35^, ^36^, ^37^, ^42^, ^43^ Despite variable manipulation, the XAI demonstrated fidelity—the ability to produce results aligned with the changes made. Additionally, testability was assessed by comparative analysis, which showed that the performance of the chosen XAI surpassed that of humans,^32^, ^33^ the black‐box AI task model,^32^ or even other XAI techniques^34^, ^42^ in terms of accuracy.

Seven (58.3%) studies^32^, ^37^, ^38^, ^39^, ^40^, ^42^, ^43^ also highlighted the plausibility of their findings derived from using XAI. These findings were plausible as they were based on valid assumptions built from established theories^32^, ^37^, ^38^, ^39^, ^40^, ^42^, ^43^ and accounted for subject variability by ensuring the samples studied were heterogeneous.^32^, ^37^, ^38^, ^39^, ^40^, ^42^, ^43^

### 3.7 Limitations of XAI use in studying cognition

Based on Figure 3, oversimplification was the most cited limitation (*n* = 11, 91.7%).^32^, ^33^, ^34^, ^35^, ^36^, ^37^, ^38^, ^39^, ^41^, ^42^, ^43^ Such oversimplification was seen in the selected XAI's input,^33^, ^34^, ^35^, ^37^, ^39^, ^41^, ^42^, ^43^ its output,^32^, ^35^, ^36^, ^37^, ^39^, ^41^, ^42^, ^43^ and the parameters or conditions^32^, ^35^, ^37^, ^38^, ^41^, ^42^ in which the XAI operated. For example, a supposedly multi‐factorial input was simplified by scaling down the volume^33^, ^35^ and types of data^34^, ^43^ fed into the XAI machinery to avoid exceeding the XAI's processing capacity. In other instances, there were insufficient collected data to begin with, preventing the XAI from performing optimally.^39^ Additionally, certain parameters or conditions had to be altered or disregarded altogether^38^ so that the experiments remained practical. For example, these experiments deviated from, or approximated at best, the actual human brain anatomy and physiology. In fact, two (16.7%) studies^32^, ^42^ operated their XAI on animal subjects such as mice and macaques. These oversimplifications of input and parameters were eventually translated into the XAI's output which were reported to be insensitive to slight data perturbations.

By extension, confounders were reported in seven (58.3%) studies.^33^, ^34^, ^35^, ^39^, ^41^, ^42^, ^43^ These confounders included gender,^41^ cultural variances,^33^ noise in imaging,^35^ varying user interpretation of XAI's findings^43^ and data collection bias.^34^, ^39^, ^42^ Also, notably, only one (8.3%) study^42^ reported inconsistencies in its XAI findings compared to evidence in the wider literature.

## 4 DISCUSSION

### 4.1 Summary of evidence

To summarize the evidence collated from the 12 included studies, XAI techniques were used to study both normal and impaired cognition. Cognitive functions studied cut across the major cognitive domains such as perceptual‐motor, social cognition, executive function, and memory domains. The types of XAI used in the 12 studies were primarily intrinsic XAI as well as attribution‐based and example‐based post hoc methods. Explainability of either a local or global scope was employed. Findings that arose from the application of these XAI techniques were anatomical and nonanatomical in nature. The anatomical findings were further delineated into neuronal, intracortical, or intercortical observations. The findings of the 12 studies were predominantly correlational. The studies either took a top‐down or bottom‐up approach in optimizing the chosen XAI for the purpose of their experiments. While the XAI techniques were lauded for their predictive power, robustness, testability, and plausibility, the reported limitations included oversimplification, the presence of confounders, and inconsistencies. Here, we discuss the significance of these findings in the wider context of the current literature.

### 4.2 Cognitive domains and XAI methods across the included studies

From the included studies, all cognitive domains, except attention, had at least some aspects examined. Perhaps, the decision to limit the scope of this review to studies published only in the last decade could explain why no included study touched on attention. However, the wider literature^44^ revealed that this cognitive domain was, in fact, extensively studied before the year 2013 through various intrinsically explainable computational methods. The extensive research before 2013 had contributed to the creation of widely accepted knowledge of attention.^16^ Over the last decade, the research community shifted focus to explore other cognitive domains as reflected in this review.

The included studies also showed that intrinsic XAI methods were used marginally more than post hoc XAI. One possible reason was that intrinsically explainable AI methods existed longer than post‐hoc methods—in fact, long before the Defense Advanced Research Project Agency (DARPA) coined the “XAI” term.^45^ DARPA started its globally renowned XAI program in 2017 which saw the development of post hoc methods.^45^ Additionally, given its simple and directly interpretable architecture,^46^ intrinsic XAI was deemed suitable in examining niche phenomena in cognitive neuroscience, especially those which involved few variables. Another possible factor influencing the choice of XAI could be cost. Either the intrinsic or post hoc XAI had the potential to be cost‐effective given the right circumstances.^47^ For instance, a task does not require a complex AI model, such as a deep neural network, if done at a small scale with minimal input. In this situation, a simple intrinsic model would be cost‐saving. However, if a complex AI task model is deemed necessary and has already been trained, adding a surrogate post hoc XAI would be cheaper than the computational and training cost of developing an intrinsic XAI from scratch.

We also identified that model‐based post hoc XAI seemed absent in the 12 included studies. This was partly due to the separation of attribution‐based and example‐based post hoc XAI from model‐based methods in our proposed XAI taxonomy. While a few other papers had labeled the former two methods as model‐agnostic post hoc XAI,^48^ we recognized that these methods were distinct in formulating and qualifying their explanations. Thus, in line with the paper by Markus,^14^ we classified them into two discrete subsets of post hoc XAI. Nevertheless, beyond the included studies, it is worth noting that the current literature also recognized other model‐based post hoc XAI^49^ like LIME and rule extraction methods like G‐REX.

### 4.3 Influence of XAI methods on the findings of the included studies

The included studies predominantly reported findings which were correlational in nature. According to Pearl's ladder of causality,^50^ there are three rungs—observations, interventions, and counterfactuals—to satisfy actual causality. Most XAI methods still operated at the first rung or based on observations, while some rose to the second rung in an attempt to establish a possible cause‐and‐effect.^50^ There had been few nascent efforts to incorporate counterfactual explanations into XAI models.^51^ Yet, some studies concluded that even the existing counterfactual algorithms for XAI are “not grounded on a causal theoretical formalism” and thus, unable to adequately prove causality.^51^, ^52^ Besides, the counterfactuals alone would not be able to definitively establish causality via the current XAI models which are prone to churning out spurious correlations.^53^ This explains why the findings from the included studies were largely correlational at best and not sufficiently causal.

Another striking observation was that intracortical findings were all derived through the application of intrinsic XAI while intercortical findings were made predominantly through post hoc XAI. This is in line with the current literature which posited that intrinsic XAI methods, while transparent, were largely restrictive in terms of explainability.^47^ Given its restrictive nature, the chosen intrinsic XAI could not draw conclusions beyond the parameters built into its system. In this case, the findings derived from intrinsic XAI methods seemed to view the cortical areas involved in isolation rather as an interconnected whole.

In addition, the included studies reported favorably on the predictive performance of their chosen XAI method regardless of the training approach being top‐down or bottom‐up. While the bottom‐up approach has the advantage of a more biologically or physiologically aligned XAI,^41^, ^42^ the top‐down approach, through a large data input, allows the XAI to perform comparatively well compared to its human counterpart in a specific task despite its physiological divergence.^32^, ^33^ Coalescing the strengths of the two approaches, some studies^54^, ^55^ beyond cognitive neuroscience, such as in image semantic segmentation, had adopted both approaches in maximizing not only the chosen XAI's predictive performance but also enhancing the interpretability or human understandability of the XAI's decisions.

### 4.4 Further evaluation of XAI used in the included studies and beyond

Although the included studies purported that their chosen XAI methods were robust as the results produced were corroborative of established theories and precedent studies, the reproducibility of these results remained unclear. The lack of XAI reproducibility was also seen outside the included studies. A recent review on the use of XAI models for electronic health records (EHRs) similarly concluded that research reproducibility was not adequately emphasized in its reviewed studies.^56^ Other studies also called for publication platforms to establish reproducibility standards as part of their publication criteria.^56^, ^57^

Despite the advantages and potential of XAI in advancing neurocognitive research, oversimplifications remained a significant sticking point as shown in the included studies and beyond. The broader literature pointed out that the oversimplifications were not only limited to the XAI's parameters, input, and output, but were also found in the XAI's explanation technique and its inherent design. For instance, the post hoc SHAP method, which produced explanations as additive contributions, was often used in the analysis of complex models that were nonadditive.^58^ For such models, the SHAP method oversimplified them, possibly leading to a false impression that the models behaved in an additive way.^59^ By extension, a study by Páez^60^ reasoned that as XAI methods were constructed with the purpose of improving the understanding of a model, they were more likely “tailored to user preferences and expectations,” rendering them more vulnerable to oversimplification and bias. Kulesza et al.^61^ also cautioned about the negative impact of oversimplifications on end users' mental models, by arguing that “when soundness was very low, user experienced more mental demand and lost trust in the explanations, thereby reducing the likelihood that users will pay attention to such explanations at all.”

### 4.5 Suggested framework for XAI in cognitive neuroscience

**Figure 4:**
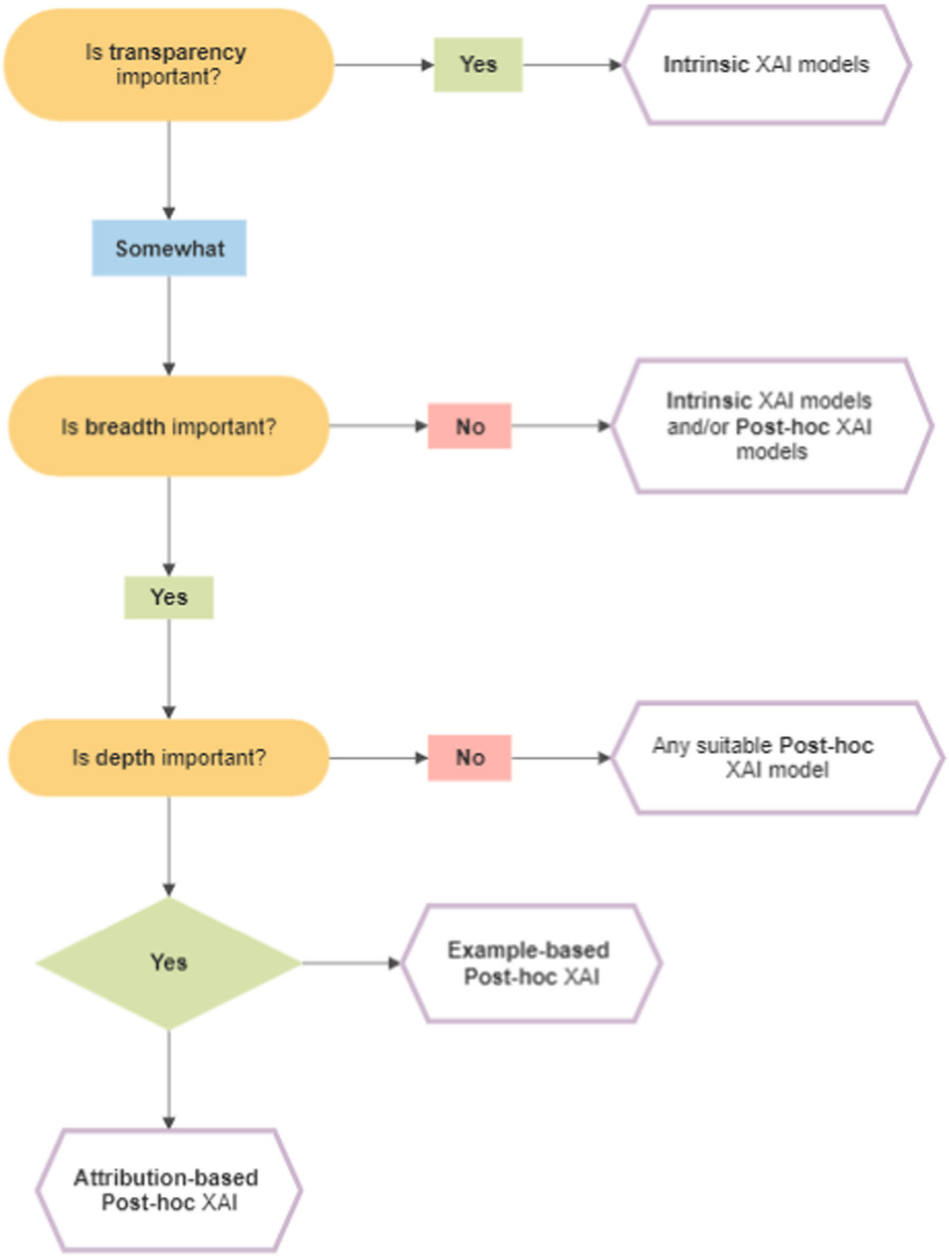
Proposed XAI Framework. This flowchart illustrates the proposed XAI framework which outlines the key factors crucial in determining the nature and quality of explanations generated by the chosen XAI. The key factors are transparency, breadth, and depth. XAI, eXplainable artificial intelligence. [Color figure can be viewed at wileyonlinelibrary.com]

Considering the salient findings within and beyond the included studies, we offer a simple framework which outlines the key factors that, in our view, are crucial in determining the nature and quality of explanations generated by the chosen XAI. The framework is by no means exhaustive and authoritative. Rather, it aims to facilitate preliminary deliberation on the current XAI options available, thereby allowing non‐AI experts in the field of cognitive neuroscience to eventually make a more informed and confident choice in their XAI methodology. Based on Figure 4, the key factors are transparency, breadth, and depth. We define transparency as the visibility of the XAI model both as a whole and its individual components. Transparency is most evident in intrinsic XAI models. Breadth, in this framework, refers to the model's capacity to identify and make sense of the interplay between entities in a broader environment of other relevant entities. A model is deemed to lack breadth if it has the tendency to consider associations in isolation of the bigger environment. The general paradigm suggests that post hoc models have breadth compared to their restrictive, intrinsic counterparts. Lastly, depth here means the ability of the model to accord its explanations a specific quality or character, rather than producing flat, nondescript correlations. For instance, depth is best illustrated by attribution‐based and example‐based post hoc models which have attempted to quantify and assign weightage to individual features involved in the explanations made.

### 4.6 Strengths and limitations of the review

We conducted a comprehensive literature search of major databases and gray literature sources which generated a substantial pool of studies. With a holistic representation of different XAI methods and the core cognitive domains, it was unlikely that the review missed out on eligible studies within its scope. A robust XAI method taxonomy, adapted from highly cited papers,^14^, ^27^, ^28^, ^29^, ^30^, ^31^ was also employed in the thematic analysis. The thematic categories were designed to minimize overlap and comprehensively cover the wide‐ranging examples of the various XAI techniques.

Nevertheless, there was subjectivity in the analysis. The contextual interpretation and classification of XAI methods and findings were dependent on the reviewer's familiarity with the subject matter. Such subjectivity, however, was minimized with two reviewers involved in the screening, data extraction, and analysis stages.

## 5 IMPLICATIONS AND FUTURE RESEARCH

Our findings are highly relevant and timely given the backdrop of increasing XAI implementation in neurocognitive experiments over recent years. These findings effectively guided the formulation of the proposed XAI framework (Figure 4) with the intention to aid the neuroscience community in their XAI methodology. Additionally, we identified potential areas for future research. Through our findings, we noted the XAI's struggle with causality and oversimplifications as well as the research community's inertia towards ensuring reproducibility in their XAI methods. Additionally, we hope that the growing interest in the application of XAI in neuroscience would allow for the uptake and iterative development of the proposed XAI framework. The future framework could consider cost and the different situations in which multiple XAI methods were employed simultaneously in a particular experiment.

## 6 CONCLUSION

The application of XAI in studying cognition is still in its infancy. The reviewed studies not only highlighted the immense potential of their chosen XAI models but also laid out their current limitations or what could be optimistically seen as teething issues. By broadly mapping the existing evidence and knowledge gaps in the literature, the findings of this scoping review could inform and steer the future development of, which all the more compels the neuroscience community to be acquainted with the tool. This review article is hopefully a step in the right direction.

## AUTHOR CONTRIBUTIONS

Shakran Mahmood and Colin Teo screened the studies and extracted data. Shakran Mahmood synthesized the findings and wrote the manuscript. Colin Teo conceptualized the idea of the review and provided insightful feedback on the manuscript. Jeremy Sim, Wei Zhang, Jiang Muyun, R. Bhuvana, Kejia Teo, Tseng Tsai Yeo, Jia Lu, Balazs Gulyas, and Cuntai Guan contributed to the study design and reviewed the manuscript.

## CONFLICT OF INTEREST STATEMENT

The authors declare no conflicts of interest.

## ETHICS STATEMENT

Not applicable.

## Supporting information

Supporting information.

## Data Availability

The data analyzed can be made available by the corresponding author upon reasonable request.

## APPENDIX A Preferred Reporting Items for Systematic Reviews and Meta‐Analyses extension for Scoping Reviews (PRISMA‐ScR) Checklist

**Table:**

Section	Item	PRISMA‐ScR checklist item	Reported on page #
Title			
Title	1	Identify the report as a scoping review.	1
Abstract			
Structured summary	2	Provide a structured summary that includes (as applicable): background, objectives, eligibility criteria, sources of evidence, charting methods, results, and conclusions that relate to the review questions and objectives.	1
Introduction			
Rationale	3	Describe the rationale for the review in the context of what is already known. Explain why the review questions/objectives lend themselves to a scoping review approach.	2–3
Objectives	4	Provide an explicit statement of the questions and objectives being addressed with reference to their key elements (e.g., population or participants, concepts, and context) or other relevant key elements used to conceptualize the review questions and/or objectives.	3
Methods			
Protocol and registration	5	Indicate whether a review protocol exists; state if and where it can be accessed (e.g., a Web address); and if available, provide registration information, including the registration number.	4
Eligibility criteria	6	Specify characteristics of the sources of evidence used as eligibility criteria (e.g., years considered, language, and publication status), and provide a rationale.	4
Information sources*	7	Describe all information sources in the search (e.g., databases with dates of coverage and contact with authors to identify additional sources), as well as the date the most recent search was executed.	4–5
Search	8	Present the full electronic search strategy for at least 1 database, including any limits used, such that it could be repeated.	3–5
Selection of sources of evidence†	9	State the process for selecting sources of evidence (i.e., screening and eligibility) included in the scoping review.	3–5
Data charting process‡	10	Describe the methods of charting data from the included sources of evidence (e.g., calibrated forms or forms that have been tested by the team before their use, and whether data charting was done independently or in duplicate) and any processes for obtaining and confirming data from investigators.	3–5
Data items	11	List and define all variables for which data were sought and any assumptions and simplifications made.	3–5
Critical appraisal of individual sources of evidence§	12	If done, provide a rationale for conducting a critical appraisal of included sources of evidence; describe the methods used and how this information was used in any data synthesis (if appropriate).	‐
Synthesis of results	13	Describe the methods of handling and summarizing the data that were charted.	3–5
Results			
Selection of sources of evidence	14	Give numbers of sources of evidence screened, assessed for eligibility, and included in the review, with reasons for exclusions at each stage, ideally using a flow diagram.	5–8
Characteristics of sources of evidence	15	For each source of evidence, present characteristics for which data were charted and provide the citations.	5–8
Critical appraisal within sources of evidence	16	If done, present data on critical appraisal of included sources of evidence (see item 12).	‐
Results of individual sources of evidence	17	For each included source of evidence, present the relevant data that were charted that relate to the review questions and objectives.	5–8
Synthesis of results	18	Summarize and/or present the charting results as they relate to the review questions and objectives.	5–8
Discussion			
Summary of evidence	19	Summarize the main results (including an overview of concepts, themes, and types of evidence available), link to the review questions and objectives, and consider the relevance to key groups.	8–12
Limitations	20	Discuss the limitations of the scoping review process.	8–12
Conclusions	21	Provide a general interpretation of the results with respect to the review questions and objectives, as well as potential implications and/or next steps.	8–12

## APPENDIX B Data Extraction Form

The main components of the data extraction form included the following:

**Table:**

Field	Options	Studies		
		Study 1	Study 2	Study 3
General				
Title	As reported in paper			
Publication year	As reported in paper			
First author	As reported in paper			
Study Aim(s)	As reported in paper			
Methods				
Study design	Case studies/correlational studies/longitudinal studies/experimental studies/clinical trial studies/mixed‐methods			
Study location	As reported in paper			
Sample size (*n*)	As reported in paper			
Target population details				
Age	As reported in paper			
Sex	Males/females/both (% composition)			
Normal cognitive Function/cognitive impairment	Provide a description as reported in paper			
Context				
Process/method of Study	Provide description of how model attempts to bridge knowledge and understanding of the cognitive function in question:			
Cognitive domain(s)/impairment(s) studied	Provide a description as reported in paper			
Concept				
Type(s) of expandable AI used	‐ Broader category: a. model‐based b. attribution‐based c. example‐based ‐ Specific name/subtype			
Outcomes of XAI use	Meets study objective? Yes/No. As reported in paper.			
Parameters to evaluate the quality of XAI(s) used				
Explanation	A system delivers or contains accompanying evidence or reason(s) for outputs and/or processes.			
Explanation accuracy/fidelity	An explanation correctly reflects the reason for generating the output and/or accurately reflects the system's process. Fulfills the following two criteria: a. The explanation describes the entire dynamic of the task model, i.e., it provides sufficient information to compute the output for a given input (**completeness/“Knowledge Limits”**). In other words, the system only operates under the conditions for which it was designed and when it reaches sufficient confidence in its output. b. The explanation is correct, i.e., it is truthful to the task model (**soundness**).			
Explanation meaningfulness/interpretability	A system provides explanations that are understandable to the intended consumer(s). Fulfills the following two criteria: a. The explanation is unambiguous, i.e. it provides a single rationale that is similar for similar instances (**clarity**). b. The explanation is not too complex, i.e., it is presented in a compact form (**parsimony**).			
Desirable features/strengths of the XAI(s) Used				
1. Robustness/generalizability:	1. In line with: – 1.1 Widely accepted theories – 1.2 Experimental data			
2. Self‐evidencing/testable/tractability:	2. Allow(s) for testability via – 2.1 Manipulation of variables – 2.2 Simulations			
3. Plausibility:	3.1 Validity of assumptions 3.2. Considers real‐world heterogeneity			
4. Predictive power	Ability to predict?			
Limitations of the XAI(s) used				
1. Contradictions/inconsistencies	1.1 Within the experiment/the study itself 1.2 Vis‐a‐vis wider literature			
2. Oversimplication	Reductionistic in terms of: 2.1 Conditions/assumptions 2.2 Input 2.3 Output			
3. Distractions	3.1 Confounding factors 3.2 Systematic bias			
Future plans/implications of the given XAI application(s)				
Other relevant findings				

The completed version of the data extraction form can be accessed via: https://docs.google.com/spreadsheets/d/1Gx7JBZLx64CuqXIFvi1WOjC-j7c8pH431KAl6nZTwmE/edit#gid=0.
