# Supplementary material for: The application of eXplainable artificial intelligence in studying cognition: A scoping review
Source: Ibrain. 2024 Sep 5;10(3):245–65. doi: 10.1002/ibra.12174 (PMC11427810; doi:10.1002/ibra.12174)
Supplement: Supplementary file 1 — Supporting information. [file IBRA-10-245-s001.docx]

**Search Strategy**

Database: Ovid MEDLINE(R) and Epub Ahead of Print, In-Process, In-Data-Review & Other Non-Indexed Citations and Daily

| **#** | **Search** | **Number of Records Identified** |
| --- | --- | --- |
| 1 | application.mp. | 447,065 |
| 2 | explainable artificial [intelligence.mp](http://intelligence.mp/). | 837 |
| 3 | explainable AI.mp. | 731 |
| 4 | explainable machine [learning.mp](http://learning.mp/). | 379 |
| 5 | XAI.mp. | 532 |
| 6 | XML.mp. | 413 |
| 7 | interpretable artificial [intelligence.mp](http://intelligence.mp/). | 53 |
| 8 | interpretable AI.mp. | 72 |
| 9 | transparent artificial [intelligence.mp](http://intelligence.mp/). | 4 |
| 10 | transparent AI.mp. | 10 |
| 11 | computational [neuroscience.mp](http://neuroscience.mp/). | 596 |
| 12 | theoretical [neuroscience.mp](http://neuroscience.mp/). | 81 |
| 13 | mathematical [neuroscience.mp](http://neuroscience.mp/). | 8 |
| 14 | study*.mp. | 3,733,311 |
| 15 | examin*.mp. | 950,849 |
| 16 | analys*.mp. | 2,578,814 |
| 17 | 2 or 3 or 4 or 5 or 6 or 7 or 8 or 9 or 10 or 11 or 12 or 13 | 2,940 |
| 18 | 14 or 15 or 16 | 5,050,418 |
| 19 | 1 and 17 and 18 | **216** |

Database: Embase

| **#** | **Search** | **Number of Records Identified** |
| --- | --- | --- |
| 1 | application.mp. | 1,166,429 |
| 2 | explainable artificial [intelligence.mp](http://intelligence.mp/). | 831 |
| 3 | explainable AI.mp. | 670 |
| 4 | explainable machine [learning.mp](http://learning.mp/). | 367 |
| 5 | XAI.mp. | 525 |
| 6 | XML.mp. | 2,293 |
| 7 | interpretable artificial [intelligence.mp](http://intelligence.mp/). | 59 |
| 8 | interpretable AI.mp. | 83 |
| 9 | transparent AI.mp. | 6 |
| 10 | transparent AI.mp. | 11 |
| 11 | computational [neuroscience.mp](http://neuroscience.mp/). | 1,479 |
| 12 | theoretical [neuroscience.mp](http://neuroscience.mp/). | 141 |
| 13 | mathematical [neuroscience.mp](http://neuroscience.mp/). | 24 |
| 14 | study*.mp. | 18,218,524 |
| 15 | examin*.mp. | 4,214,477 |
| 16 | analys*.mp. | 11,427,472 |
| 17 | 2 or 3 or 4 or 5 or 6 or 7 or 8 or 9 or 10 or 11 or 12 | 5,744 |
| 18 | 13 or 14 or 15 | 22,168,742 |
| 19 | 1 and 16 and 17 | **540** |

Database: Cochrane Central

| **#** | **Search** | **Number of Records Identified** |
| --- | --- | --- |
| 1 | Application: ti,ab,kw (Word variations have been searched) | 101 657 |
| 2 | explainable artificial intelligence | 14 |
| 3 | explainable AI | 12 |
| 4 | explainable machine learning | 16 |
| 5 | XAI | 19 |
| 6 | XML | 60 |
| 7 | interpretable artificial intelligence | 9 |
| 8 | interpretable AI | 18 |
| 9 | transparent artificial intelligence | 17 |
| 10 | transparent AI | 45 |
| 11 | computational neuroscience | 178 |
| 12 | theoretical neuroscience | 209 |
| 13 | mathematical neuroscience | 45 |
| 14 | studying | 6518 |
| 15 | examination | 78 309 |
| 16 | analysis | 566 752 |
| 17 | 2 or 3 or 4 or 5 or 6 or 7 or 8 or 9 or 10 or 11 or 12 or 13 | 591 |
| 18 | 14 or 15 or 16 | 624 140 |
| 19 | 1 and 17 and 18 | **337** |

Database: Web of Science

| **#** | **Search** | **Number of Records Identified** |
| --- | --- | --- |
| 1 | ALL=(Application) | 5 829 541 |
| 2 | ALL=(explainable artificial intelligence) | 5458 |
| 3 | ALL=(explainable AI) | 4982 |
| 4 | ALL=(explainable machine learning) | 5207 |
| 5 | ALL=(XAI) | 3132 |
| 6 | ALL=(XML) | 23 354 |
| 7 | ALL=(interpretable artificial intelligence) | 3535 |
| 8 | ALL=(interpretable AI) | 2385 |
| 9 | ALL=(transparent artificial intelligence) | 2317 |
| 10 | ALL=(computational neuroscience) | 22 433 |
| 11 | ALL=(theoretical neuroscience) | 8138 |
| 12 | ALL=(mathematical neuroscience) | 8625 |
| 13 | #2 OR #3 OR #4 OR #5 OR #6 OR #7 OR #8 OR #9 | 38 477 |
| 14 | #10 OR #11 OR #12 | 34 136 |
| 15 | #1 AND #13 AND #14 | **36** |
